# Simultaneous integrated boost concepts in definitive radiation therapy for esophageal cancer: outcomes and toxicity

**DOI:** 10.1186/s13014-021-01749-x

**Published:** 2021-02-01

**Authors:** J. Ristau, M. Thiel, S. Katayama, I. Schlampp, K. Lang, M. F. Häfner, K. Herfarth, J. Debus, S. A. Koerber

**Affiliations:** 1 Department of Radiation Oncology, Heidelberg University Hospital, Im Neuenheimer Feld 400, 69120 Heidelberg, Germany; 2 Department of Radiation Oncology, Heidelberg Institute of Radiation Oncology (HIRO), Heidelberg University Hospital, Heidelberg, Germany; 3 National Center for Tumor Diseases (NCT), Heidelberg University Hospital, Heidelberg, Germany; 4 Clinical Cooperation Unit Radiation Oncology, German Cancer Research Center (DKFZ), Heidelberg, Germany; 5 Department of Radiation Oncology, Heidelberg Ion-Beam Therapy Center (HIT), Heidelberg University Hospital, Heidelberg, Germany; 6 Core Center Heidelberg, German Cancer Consortium (DKTK), Heidelberg, Germany; 7 Radiation Oncology Unit Speyer, Speyer, Germany

**Keywords:** Esophageal cancer, Chemoradiation, IMRT, Simultaneous integrated boost

## Abstract

**Background:**

Radiation therapy and chemoradiation therapy play a major role in the definitive management of esophageal cancer. Survival in esophageal cancer patients is still relatively poor, mostly due to high rates of local recurrence and distant metastases. It is hypothesized that dose escalation in radiotherapy could improve outcomes. Therefore, this retrospective analysis aimed to investigate the outcomes and toxicity in patients treated with local dose escalation by means of using simultaneous integrated boost concepts.

**Methods:**

Between 2012 and 2018, 101 patients with esophageal carcinoma were analyzed in this monocentric, retrospective study. All patients received definitive chemoradiation or radiation therapy alone as intensity modulated radiotherapy. The prescribed dose was 50.4 Gy in 28 fractions to the primary tumor and the elective lymph nodes as well as a simultaneous integrated boost (SIB) with 58.8 Gy to macroscopic tumor and lymph node metastases. Endpoints were overall survival (OS), progression free survival (PFS), local control rate (LCR) and toxicity.

**Results:**

60 patients (59.4%) received chemoradiation, 41 patients (40.6%) radiotherapy alone. The median follow up was 17 months (range 0–75 months). OS, PFS and LCR were at 63.9%, 53.9% and 59.9% after 1 year and 37.6%, 34.5% and 36.1%, respectively after 3 years. 16 patients (15.8%) in total developed a locoregional recurrence within the field of radiation. In 48 patients (47.5%) at least one grade III° (CTCAE) toxicity was documented during radiotherapy, mostly dysphagia (36 pat., 75%). One patient suffered from a grade IV° pneumonia.

**Conclusion:**

This retrospective analysis demonstrates that a SIB concept in definitive (chemo)radiation therapy is safe and feasible, showing acceptable outcomes in this patient cohort. Considering that this cohort mainly consists of elderly patients not eligible for chemotherapy in many cases, we emphasize the aspect of SIB radiation therapy as potential partial compensation for omitted simultaneous chemotherapy. Prospective studies are needed for validation.

## Introduction

Globally, esophageal cancer is ranked seventh and sixth in terms of cancer incidence and overall mortality, respectively, with approximately 70% of all cases occurring in men and a majority of all cases in less-developed countries [1]. In 2018, there were 572.000 new cases and 509.000 associated deaths [1]. The two major histological types of esophageal cancer, adenocarcinoma (AC) and squamous cell carcinoma (SCC), differ greatly in terms of their etiologic and epidemiologic risk factors. Whereas SCC is most common in South-Eastern and Central Asia, the highest incidence of AC can be observed in well-developed regions such as Northern and Western Europe and Northern America [2]. SCC being the predominant subtype, incidence rates have been decreasing in high-income countries in recent years, potentially due to a decline in tobacco smoking, one of the major risk factors. On the other hand, AC incidence rates have been rising in western populations, which has been attributed to obesity and reflux [3, 4]. As more than two thirds of all patients are diagnosed with locally advanced or even metastasized disease, 5-year-overall survival is relatively poor, ranging from 15 to 25% [5]. Treatment of esophageal cancer depends greatly on clinical tumor stage and regularly implies multidisciplinary assessment. Patients with resectable locally advanced esophageal or esophagogastric junctional cancer benefit from addition of neoadjuvant chemoradiotherapy with improved overall survival compared to surgery alone [6–8]. There is only few data comparing treatments involving surgery with definitive chemoradiation therapy (dCRT). A meta-analysis with mainly thoracic SCC showed no difference in overall survival with higher loco-regional progression rates in patients receiving dCRT but less treatment-related mortality compared to surgically treated patients [9]. In case of non-operability, definitive chemoradiation therapy plays a major role for both locally advanced AC and SCC, achieving five-year overall survival rates between 10 and 35% [10, 11]. In the definitive setting, the combination of chemotherapy and radiotherapy is more effective than radiotherapy alone [12, 13]. Total radiation doses generally range between 50 and 60 Gy. Several studies have concluded that total doses of more than 60 Gy can be applicated safely [9, 14–16], however, there is no clear evidence for a benefit of dose-escalation. The only published randomized trial on dose escalation did not increase survival or local/regional control [17]. It has been criticized, though, as higher treatment-related mortality rates were shown in the high-dose radiation arm but mostly before patients reached 50.4 Gy, which may be due to imbalanced prognostic factors. Preliminary data of the recent ARTDECO-trial, however, indicate that local control rates could not be improved using dose escalation up to 61.6 Gy [18]. Retrospective data suggest a dose–effect-correlation [19] with acceptable outcomes for patients receiving doses of 60–70 Gy [20, 21]. On the other hand, clinical routine demonstrates that older patients with comorbidities are often at risk for severe side-effects and therefore precluded from combined treatment approaches [22, 23]. Obviously, clinical outcome in daily practice is significantly altered by these factors, which is often not taken into account in clinical trials as those patients are generally underrepresented [24, 25]. A practical clinical approach to address the issue of dose-escalation for regions at risk of local failure could be the concept of simultaneous integrated boost (SIB) application. Several studies have demonstrated that SIB usage is safe and feasible with acceptable toxicities [26–28]. Therefore, this retrospective study aims to give a realistic overview of a large cohort of esophageal cancer patient treated with dCRT with simultaneous boost concepts.

## Material and methods

Before data retrieval, this single-institutional, retrospective study was approved by the local ethics committee (S-190/2018).

### Patient population

Patient selection was based on a retrospective database query of the department of radiation oncology at the Heidelberg University Hospital. Patients of any age who received definitive local radiation or chemoradiation therapy for esophageal cancer (adenocarcinoma or squamous cell carcinoma) of any stage with curative or palliative intention between 11/2012 and 07/2018 were included in this analysis. Patients managed with neoadjuvant treatment concepts consisting of radiotherapy or chemoradiation followed by surgery were not included. Other exclusion criteria were chemotherapy or immunotherapy without irradiation, radiotherapy of metastases, other simultaneous malignancies or history of malignancies within three years before therapy, death before start of planned radiotherapy, or incomplete data. Also, type II and III adenocarcinomas of the gastroesophageal junction and patients with distant metastases except of supraclavicular lymph node metastases were not eligible for this study. For a total of 101 patients who met these criteria, clinical data were extracted from the clinic’s patient data management system and electronic archives. Toxicity was documented according to the Common Toxicity Criteria for Adverse Events (CTCAE) version 5.0.

### Treatment

All radiation therapy concepts were based on CT-planned intensity-modulated radiotherapy (IMRT) as helical IMRT (Tomotherapy^®^) at the Heidelberg University Hospital. PET/CT-imaging was not part of the routine diagnostics. The prescribed dose for all 101 patients was 50.4 Gy with a simultaneous integrated boost up to a median total dose of 58.8 Gy in 28 fractions. The median single doses were 1.8 Gy and 2.1 Gy (SIB), respectively, being the institutional standard. The gross tumor volume (GTV) included all macroscopic tumor visible on the planning CT, including suspected nodal disease. The clinical target volume (CTV) was created by adding margins to the GTV (radial 0.5–1 cm, craniocaudal: 4–5 cm) and adjusted for the lymphatic drainage areas within this expansion. In cervical or proximal tumors, supraclavicular nodes were included. Also, celiac nodes were defined as CTV in case of distal tumor location. Another margin of 0.5–1 cm was added to create the planning target volume (PTV). In case of dose escalation in dCRT, the boost CTV was derived from the initial GTV by addition of a 1.5–2 cm craniocaudal and a 0.5–1 cm radial margin. The margin from CTV to PTV for the boost was also 0.5–1 cm. Concomitantly used chemo-/immunotherapeutic regimes included cisplatin/5-FU, FOLFOX and cetuximab.

### Follow-up

The median follow up for all patients was 17 months. Routinely, patients underwent CT scans every 3 months for the first two years after therapy and every 6 months thereafter. Endoscopic control examinations were scheduled for the first follow-up after (chemo-)radiation therapy and in case of suspicious findings in other follow-up CT scans or new clinical symptoms suggesting progressive disease.

### Statistics

Statistical endpoints that were examined in this study included overall survival (OS), progression-free survival (PFS) and locoregional control rate (LCR). Starting dates were defined as the start of therapy. PFS was defined as the time to progressive disease, whereas LCR was defined as time to local recurrence within the former field of radiation accounting for both recurrence of primary tumors or lymph node metastases. Recurrences inside or outside the SIB volume were also documented separately.

The Kaplan–Meier method was applied to estimate OS, PFS and LCR. Log-rank tests were used for univariate analyses of therapy-associated parameters and patient characteristics. For multivariate analysis of relevant clinical factors, cox-regression was used. For all tests, a *P* value of < 0.05 was considered statistically significant. As this was an exploratory analysis, no adjustments for multiple comparisons were performed. For all statistical analyses, SPSS version 25 (IBM) was used.

## Results

Patient characteristics are listed in Table 1. The median patient age was 72 years (range 36–87 years). More than 75% were male. Most patients had locally advanced disease at the time of diagnosis. 77.2% of all patients had lymph node metastases, 10.9% of all lymph node metastases being localized in supraclavicular position. Location of the primary tumor (defined by its proximal edge) was cervical in 12.9%, upper thoracic in 22.8%, middle thoracic in 19.8% and lower thoracic/abdominal in 44.6%. Tumor histology was squamous cell carcinoma (SCC) in 75.2% and adenocarcinoma (AC) in 23.8% (one patient had an adenosquamous carcinoma). Prior to radiotherapy, nutritional support with parenteral nutrition or via percutaneous endoscopic gastrostomy (PEG) was necessary in 6.9 and 9.9% of all patients. In the course of radiation therapy, the proportion of patients in need of parenteral nutrition or PEG increased to 30.7% and 38.6%, respectively.

**Table 1:** Patient characteristics

	n	(%)
Number of patients	101	(100)
*Sex*		
Male	76	(75.2)
Female	25	(24.8)
*Age at start of therapy (median, range)*	72 y (36–87)	
< 50 y	4	(4.0)
50–75 y	64	(63.4)
> 75 y	33	(32.7)
*Karnofsky performance status scale*		
> 80%	23	(22.8)
≤ 80%	78	(77.2)
Median (range)	70%	(50–100%)
Simultaneous chemoradiation therapy	60	(59.4)
*Tumor histology*		
squamous cell carcinoma	76	(75.2)
adenocarcinoma	24	(23.8)
other	1	(1.0)
*Grading*		
Gx	11	(10.9)
G1	4	(4.0)
G2	54	(53.5)
G3	32	(31.7)
*T-stage*		
T1	4	(4.0)
T2	9	(8.9)
T3	53	(52.5)
T4	22	(22.8)
T2 +	13	(12.9)
*Tumor extension*		
< 5 cm	39	(38.6)
5–20 cm	57	(56.4)
> 20 cm	5	(5.0)
*N-stage*		
N0	23	(22.8)
N +	78	(77.2)

5.0% of all patients had undergone chemotherapy prior to definitive (chemo)-radiation, 4.0% other therapeutic interventions such as mucosectomy and 2.0% had an insertion of an esophageal stent. More than half of the patients were current or former smokers and/or alcohol consumers on a regular basis. The median Charlson Comorbidity Index was 4 points. Regarding diagnostic procedures prior to treatment, all patients underwent esophagogastroduodenoscopy (EGD), added by endosonography in 18.8%. Furthermore, 14.9% of all patients had FDG-PET/CT imaging.

60 patients (59.4%) received a definitive chemo-radiation therapy. 41 patients (40.6%) had sole radiation therapy, mostly due to comorbidities or poor general health status. Systemic treatment regimes were cisplatin/5-fluoruracil in 33.7%, FOLFOX in 23.8% and cetuximab in 2.0%.

### Survival

**Fig. 1:**
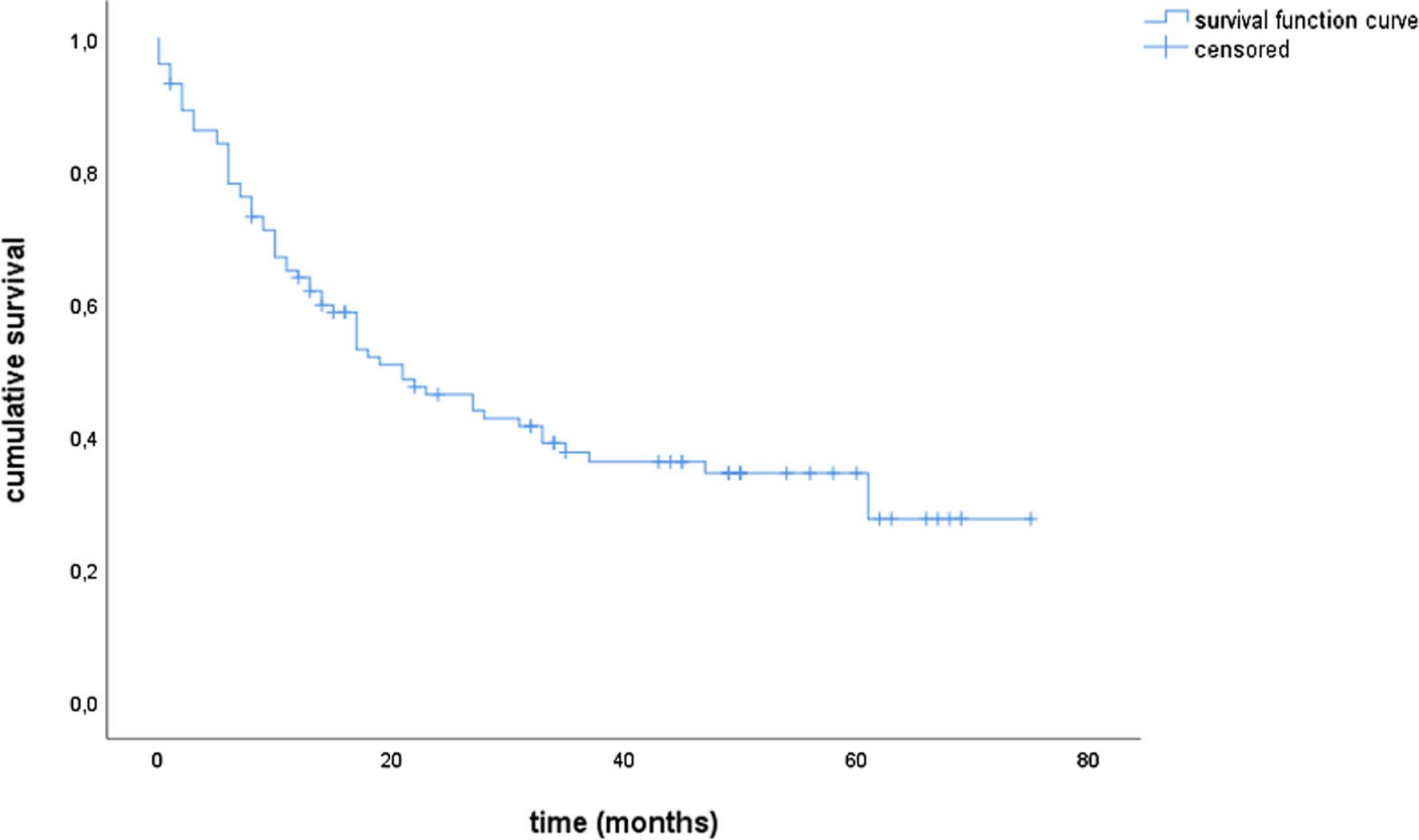
Overall survival

**Fig. 2:**
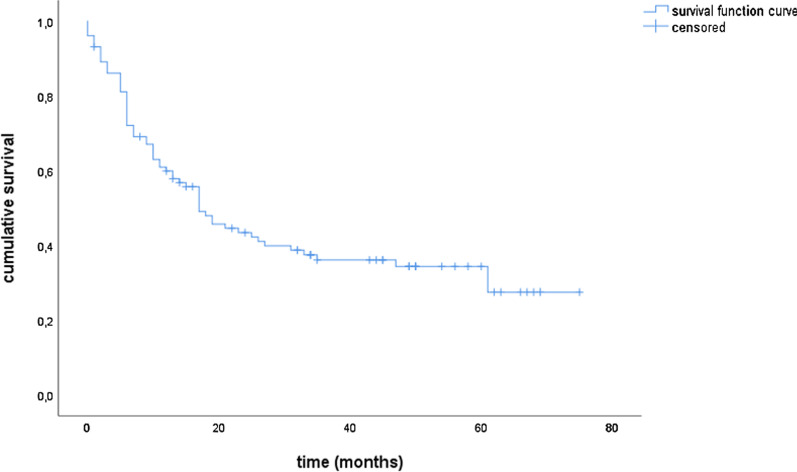
Progression-free survival

**Fig. 3:**
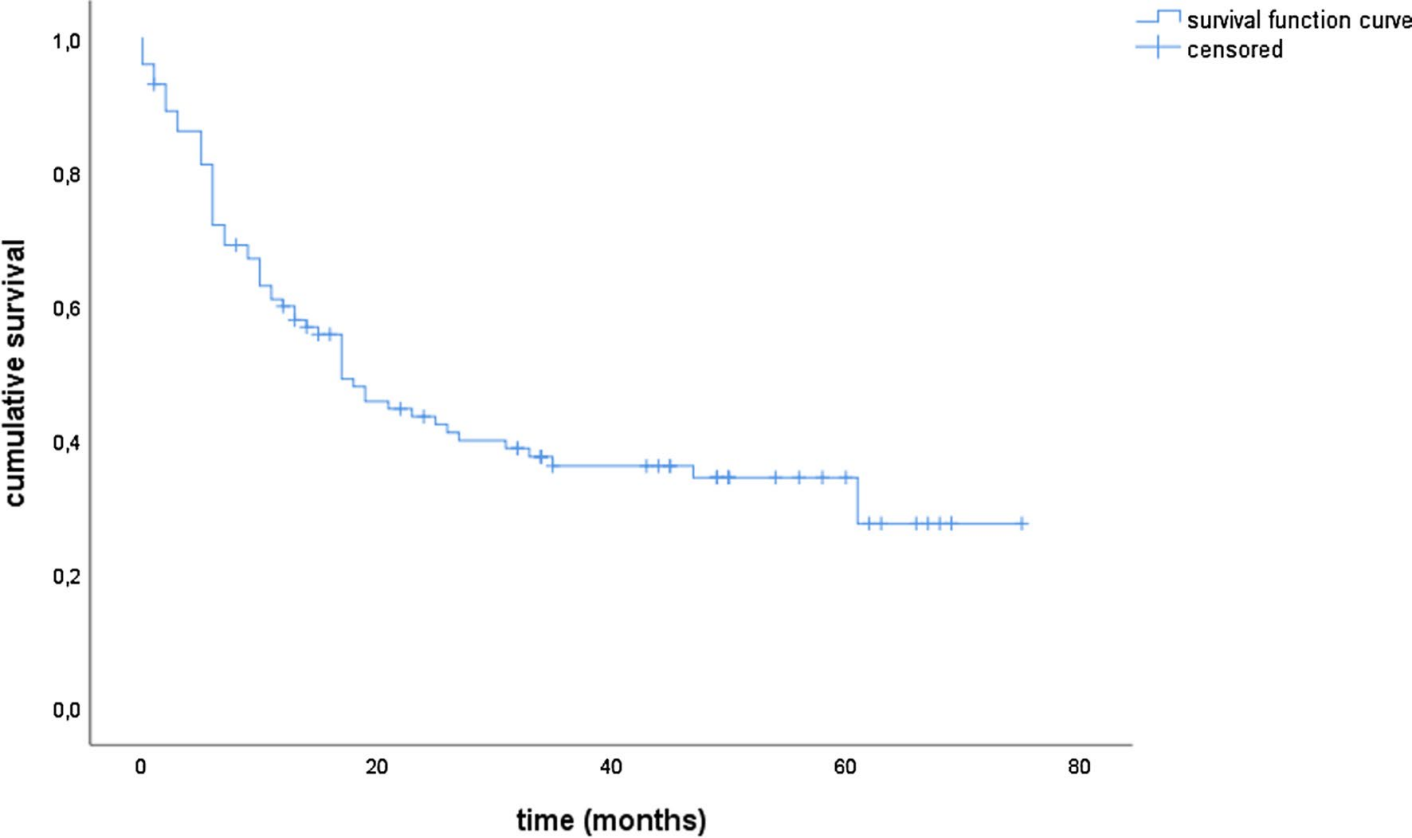
Locoregional control

Median OS and PFS for the entire cohort were 21.0 and 15.0 months, respectively. The estimated 1- and 3-year survival rates were 63.9% and 37.6% for OS and 53.9% and 34.5% for PFS, respectively (Figs. 1 and 2). The locoregional control rate was 59.9% after one year and 36.1% after 3 years (Fig. 3). The median locoregional recurrence free survival was 17.0 months (95% CI 11.6–22.4).

In total, 16 patients developed a locoregional failure. 9 of these patients did not have concomitant chemotherapy. In 15 patients, the recurrence was localized within the simultaneous integrated boost volume.

Patients ≤ 75 years of age had a significantly better OS compared to patients older than 75 years (*p* = 0.048). Older age was also a significant risk factor for worse PFS in multivariate analysis (MVA, Table 2). The application of concurrent chemotherapy was strongly associated with longer OS, PFS and LCR (p < 0,001 each). This benefit was also found in MVA, but not for PFS. Univariate analysis of body mass index (BMI) revealed that patients with underweight (BMI < 18.5) had a significantly shorter PFS (*p* = 0.027, Tables 2 and 3) which was confirmed in MVA for both PFS and LCR.

**Table 2:** Multivariate analyses

		OS			PFS		
Parameter	Reference	*p* value	HR	95% confidence interval	*p* value	HR	95% confidence interval
*T-stage*							
T3/4	T1/2	0.633	0.8	0.3–2.1	0.454	0.7	0.3–1.8
T2 + ^1^	T1/2	0.388	0.6	0.2–1.9	0.346	0.6	0.2–1.8
Chemotherapy	No chemotherapy	0.017	0.4	0.2–0.8	0.070	0.5	0.2–1.1
Charlson index	No comorbidities	0.897	1.1	0.5–2.5	0.647	1.2	0.5–2.8
Age		0.060	1.0	1.0–1.1	0.049	1.1	1.0–1.1
*BMI*							
Underweight	Normal weight	0.062	2.5	1.0–6.7	0.004	3.9	1.5–9.8
Overweight		0.426	1.3	0.7–2.5	0.530	1.2	0.6–2.4
Obese		0.978	1.0	0.4–2.2	0.680	1.2	0.5–2.7

^1^T2 + was used for tumors stages at least T2 but without further exact T-staging

**Table 3:** Univariate analyses

		OS			PFS		
Parameter	Number of patients	Events	Median 95% confidence interval	*p* value (log rank test)	Events	Median 95% confidence interval	*p* value (log rank test)
T-Stage				0.766			0.585
T1	4	3	0.0–51.3		3	0.0–51.3	
T2	9	5	0.0–58.1		6	0.0–14.8	
T3	53	31	9.0–33.0		31	3.9–30.1	
T4	22	16	6.6–23.4		17	0.0–13.4	
T2 + ^1^	13	8	25.9–48.1		8	4.8–33.2	
Chemotherapy				< 0.001			< 0.001
Yes	60	30	15.4–78.6		32	0.0–58.9	
No	41	33	5.9–12.1		33	2.9–9.1	
Charlson index				0.096			0.089
Score 2	20	9	-		9	-	
Score > 2	81	54	10.1–25.9		56	8.3–21.7	
Age				0.036			0.092
≤ 75 years	68	38	14.4–41.6		40	6.9–27.1	
> 75 years	33	25	5.6–16.4		25	0.0–14.7	
BMI							0.027
underweight	8	7	0.0–20.1		8	2.3–7.7	
normal weight	43	26	6.8–47.2		26	11.9–26.2	
overweight	29	19	6.0–28.0		19	4.1–25.9	
obese	21	11	0.0–44.0		12	0.0–35.4	

^1^T2 + was used for tumors stages at least T2 but without further exact T-staging

### Toxicity

A list of treatment-related toxicities is supplied in Table 4. In 1 patient, radiotherapy had to be paused for more than 3 days due to treatment-related toxicity, in 3 other patients due to other reasons. Therapy was aborted in 7 patients (6.9%) due to other medical reasons, 4 of these patients died during the projected time of treatment. De-escalating modifications to chemotherapy treatments were necessary in 8 patients (7.9%). Concomitant chemotherapy had to be interrupted or terminated in 20 cases (19.8%). Acute toxicity was manageable with grade 3 toxicities seen in 48/101 patients (47.5%) and one grade 4 pneumonia (1.0%), grade 3 being mostly dysphagia and nausea/emesis (see Table 4). Prior to start of therapy, grade III symptoms were present in 20 patients (19.8%). One patient died due to esophageal bleeding after a PEG had been placed in direct puncture technique, another died due to severe pneumonia with consecutive septic shock.

**Table 4:** Toxicity

	Acute (n = 101)					Subacute (< 3 months) (n = 82)					> 3 months (n = 72)				
Grading	G1	G2	G3	G4	G5	G1	G2	G3	G4	G5	G1	G2	G3	G4	G5
*Toxicity (%)*															
Dysphagia	25.7	30.7	35.6	0	0	30.5	22.0	9.8	0	0	40.3	26.4	27.8	5.6	0
Anorexia	0	3.0	6.9	0	0	0	0	1.2	0	0	0	1.4	2.8	0	0
Nausea/emesis	18.8	18.8	14.9	0	0	6.1	4.9	2.4	0	0	12.5	1.4	1.4	0	0
Pulmonary toxicity	5.0	12.9	4.0	0	0	19.5	17.1	0	0	0	36.1	26.4	1.4	0	0
Fistula	0	0	0	0	0	0	0	0	0	0	1.4	0	0	0	0
Strictures/bouginage necessary	0	0	0	0	0	0	0	0	0	0	0	0	19.4	0	0
Bleeding	1.0	0	0	0	1.0	0	1.2	0	0	0	0	1.4	1.4	0	0
Dermatitis	23.8	12.9	2.0	0	0	6.1	1.2	0	0	0	0	0	0	0	0
Weight loss	29.7	14.9	0	0	0	11.0	3.7	0	0	0	5.6	5.6	0	0	0
Diarrhea	74.3	19.8	5.9	0	0	2.4	1.2	0	0	0	2.8	0	0	0	0
Fatigue	19.8	18.8	3.0	0	0	13.4	6.1	3.7	0	0	26.4	9.7	2.8	0	0
Cardiac toxicity	n/a					3.7	1.2	1.2	0	0	5.6	1.4	0	0	0

70 patients (69.3%) required assistance with nutrition in the form of PEG or TPN (total parenteral nutrition). Regarding late toxicities, 14 patients (19.4% of 72 documented follow-ups) needed bouginage of the esophagus as a result of strictures or stenosis. 27.8% of the patients still suffered from grade 3 dysphagia after more than 3 months from the end of treatment. Dysphagia, weight loss, nausea/emesis, radiation dermatitis and fatigue were the most common toxicities irrespectively whether patients were older or younger than 75 years (Additional file 1: Table S1).

## Discussion

In this retrospective monocentric study we have analyzed a cohort of patients with esophageal cancer treated with definitive chemoradiation or radiotherapy alone. To our knowledge, it represents one of the largest cohorts treated with definitive radiation concepts with SIB. As many prospective trials explicitly require a certain general state of health prior to inclusion, those studies may not always reflect the actual clinical situation. Retrospective analyses naturally depend on precise documentation and consistent quality of data, therefore interpretation of results can often be challenging. Nevertheless, by including patients independent of their Karnofsky index, age and pre-existing comorbidities, we reflect normal clinical routine, providing important information about treatment outcomes and toxicity in the non-surgical management of esophageal cancer patients. Gender distribution with a majority of male patients in our cohort reflects the global status [1]. The proportion of squamous cell carcinoma in our cohort (75.2%) was larger than literature suggested for a German population [29], probably due to the fact that the German guidelines recommend surgery-involving treatment concepts for adenocarcinoma if technically feasible [30].

The use of IMRT with SIB has been shown to be safe and effective in previous studies. A dosimetric analysis by Welsh et al. demonstrated that doses to primary tumors could be increased by 28% compared to 2D-conformal radiotherapy techniques [31]. Fu et el. reported on a significantly reduction of doses to normal tissue using SIB-IMRT compared with 3D-conformal radiotherapy and sequential boost application [32]. A phase II study by Yu et al. was able to show slightly longer survival times compared to our cohort with 3-year-OS, PFS and LCR rates of 42.2%, 40.7% and 67.5%, respectively [27]. Besides different definitions of LCR, one reason for this difference could be the use of higher SIB doses of 63 Gy. The significance of patient selection is well-reflected in a recent study of Li et al. including patients only up to an age of 70 years with Karnofsky indices of at least 70%, resulting in 1-year OS and LCR rates of 76.9% and 78.8% [28]. Patients in our cohort were older than most cohorts previously published with a median age of 72 years, reflecting the actual clinical challenge in many western countries facing demographic changes towards growing proportions of elderly people. Considering that the number of elderly cancer patients in general is likely to increase in the future, treatment decisions involving chemoradiation therapy will have to carefully take into account patients’ comorbidities, functional status and expected treatment-related toxicities. In a large proportion of patients in this study, simultaneous chemotherapy application was not feasible due to comorbidities. Therefore, it is clear that our results have to be interpreted carefully as sole definitive radiation therapy is inferior to dCRT [12, 33], which has recently also been shown for elderly patients [34]. We assume that the relatively low local control rates in our cohort can be explained by the age distribution and patients’ general health status. Our results underline the need for modified treatment concepts in elderly patients, as older age was significantly associated with worse survival. In almost all patients who had locoregional failure in our study, the localization of local progression was inside the SIB volume. More than half of these patients were not eligible for simultaneous chemotherapy. In our opinion, this could indicate that SIB doses of 58.8 Gy are not high enough to compensate for omitted chemotherapy. Dose escalation of more than 60 Gy thus might be appropriate for patients unable to receive systemic therapies. Data generated by Conroy et al. comparing different chemotherapeutic drugs along with definitive radiation therapy without use of SIB show worse 3-year OS and PFS rates with 26.9% and 17.4% [35]. Another large retrospective study analyzing dCRT without SIB resulted in better 3-year OS, PFS and LCR with 39.9%, 33.6% and 35.3%, which might be due to a higher median radiation dose of 60 Gy [20]. A recent prospective phase I/II study showed superior overall survival and local control for a small cohort that received chemoradiation with SIB compared to a standard-dose cohort [36]. Toxicity was less compared to our study, assumably due to the lower median age of 65 years.

The relatively high overall rate of grade III toxicity in our study cohort is primarily based on the rate of acute grade III dysphagia in our cohort, which was significant with 35.6%, but manageable, considering that 12.9% of all patients already suffered from tumor-associated grade III dysphagia prior to therapy (data not shown). Prospective data of two large studies investigating dose escalation in dCRT of esophageal cancer patients (NCT01348217; NCT02556762) will add valuable information to the issue of the optimal radiation dose for these patients, which remains inconclusive. The large British phase II/III SCOPE2 trial (NCT02741856) is combining neoadjuvant chemotherapy and PET/CT-based response assessment as well as the aspect of dose intensification up to 60 Gy in a 2 × 2 design, which will hopefully help improve treatment in the nonsurgical approach of esophageal cancer.

## Conclusion

We were able to demonstrate that the feasibility of SIB dose escalation to areas of high risk of local failure in a large cohort of esophageal cancer patients. Considering the general health status of the patients in our cohort, survival rates are acceptable and toxicity was moderate. As addition of chemotherapy is often precluded in these patients, we hypothesize that further selective dose escalation could help reduce the rate of recurrences. In particular, in cases where chemotherapy cannot be applied due to comorbidities or elderly patients, SIB radiation therapy could at least in parts compensate for omitted simultaneous chemotherapy. Prospective trials involving SIB concepts are needed to further evaluate the potential oncological benefit in these specific groups of patients.

## Supplementary Information

**Additional file 1.** **Table S1:** Toxicity by age.

## Data Availability

The datasets used and/or analysed during the current study are available from the corresponding author on reasonable request.
