# Supplementary material for: Simultaneous integrated boost concepts in definitive radiation therapy for esophageal cancer: outcomes and toxicity
Source: Radiat Oncol. 2021 Feb 1;16:23. doi: 10.1186/s13014-021-01749-x (PMC7852221; doi:10.1186/s13014-021-01749-x)
Supplement: Supplementary file 1 — Additional file 1. Table S1: Toxicity by age. [file 13014_2021_1749_MOESM1_ESM.docx]

| Table S1: Toxicity by age  Toxicity (%) | | | | | | | | | | | | | | |
| --- | --- | --- | --- | --- | --- | --- | --- | --- | --- | --- | --- | --- | --- | --- |
|  | Acute | | | |  | Subacute (< 3 months) | | | |  | > 3 months | | | |
|  | ≥ 75 (n=40) | | ≤ 74 y (n=61) | |  | ≥ 75 y (n=33) | | ≤ 74 y (n=49) | |  | ≥ 75 y (n=28) | | ≤ 74 y (n=44) | |
| Grading | G1/G2 | ≥ G3 | G1-G2 | ≥ G3 |  | G1/G2 | ≥ G3 | G1/2 | ≥ G3 |  | G1/G2 | ≥ G3 | G1/2 | ≥ G3 |
| anorexia | 2.5  (1/40) | 10.0  (4/40) | 3.3  (2/61) | 4.9  (3/61) |  | 0 | 0 | 0 | 2.0 (1/49) |  | 3.6  (1/28) | 0 | 0 | 4.5 (2/44) |
| dysphagia | 60.0  (24/40) | 35.0  (14/40) | 50.8 (31/61) | 36.0 (22/61) |  | 54.5 (18/33) | 0 | 51.0 (25/49) | 16.3 (8/49) |  | 46.4 (13/28) | 0 | 59.1 (26/44) | 4.5 (2/44) |
| nausea/  emesis | 37.5  (15/40) | 5.0  (2/40) | 37.7 (23/61) | 21.3 (13/61) |  | 9.1  (3/33) | 0 | 12.2 (6/49) | 4.0 (2/49) |  | 10.7 (3/28) | 0 | 15.9 (7/44) | 2.3 (1/44) |
| Pulmonary toxicity | 20.0  (8/40) | 2.5  (1/40) | 31.1 (19/61) | 8.2  (5/61) |  | 18.2 (6/33) | 0 | 16.3 (8/49) | 0 |  | 39.3 (11/28) | 0 | 27.3 (12/44) | 0 |
| fistula | 0 | 0 | 0 | 0 |  | 0 | 0 | 0 | 0 |  | 3.6  (1/28) | 0 | 0 | 0 |
| Strictures/bouginage necessary | 0 | 0 | 0 | 0 |  | 0 | 0 | 0 | 0 |  | 0 | 17.8 (5/28) | 0 | 20.5 (9/44) |
| bleeding | 2.5  (1/40) | 0 | 0 | 1.6  (1/61) |  | 3.0  (1/33) | 0 | 0 | 0 |  | 3.6  (1/28) | 0 | 0 | 0 |
| dermatitis | 32.5  (13/40) | 0 | 39.3 (24/61) | 1.6  (1/61) |  | 6.1  (2/33) | 0 | 8.2  (4/49) | 0 |  | 0 | 0 | 0 | 0 |
| Weight loss | 50.0  (20/40) | 0 | 41.0 (25/61) | 0 |  | 15.2 (5/33) | 0 | 14.3  (7/49) | 0 |  | 7.1  (2/28) | 0 | 13.6 (6/44) | 0 |
| diarrhea | 22.5  (9/40) | 0 | 27.9 (17/61) | 0 |  | 3.0  (1/33) | 0 | 4.1  (2/49) | 0 |  | 3.6  (1/28) | 0 | 2.3  (1/44) | 0 |
| fatigue | 37.5  (15/40) | 5.0  (2/40) | 39.3 (24/61) | 1.6  (1/61) |  | 33.3 (11/33) | 6.1 (2/33) | 10.2 (5/49) | 2.0 (1/49) |  | 50.0 (14/28) | 3.6 (1/28) | 27.3 (12/44) | 2.3 (1/44) |
| cardiac toxicity | 0 | 0 | 0 | 0 |  | 6.1 (2/33) | 0 | 6.1  (4/49) | 2.0 (1/49) |  | 14.3 (4/28) | 0 | 2.3  (1/44) | 0 |
